# Altered Neurocircuitry in the Dopamine Transporter Knockout Mouse Brain

**DOI:** 10.1371/journal.pone.0011506

**Published:** 2010-07-09

**Authors:** Xiaowei Zhang, Elaine L. Bearer, Benoit Boulat, F. Scott Hall, George R. Uhl, Russell E. Jacobs

**Affiliations:** 1 Biological Imaging Center, Beckman Institute, California Institute of Technology, Pasadena, California, United States of America; 2 Department of Pathology, University of New Mexico Health Sciences Center, Albuquerque, New Mexico, United States of America; 3 Molecular Neurobiology Branch, Intramural Research Program, National Institute on Drug Abuse, Baltimore, Maryland, United States of America; INSERM U901, France

## Abstract

The plasma membrane transporters for the monoamine neurotransmitters dopamine, serotonin, and norepinephrine modulate the dynamics of these monoamine neurotransmitters. Thus, activity of these transporters has significant consequences for monoamine activity throughout the brain and for a number of neurological and psychiatric disorders. Gene knockout (KO) mice that reduce or eliminate expression of each of these monoamine transporters have provided a wealth of new information about the function of these proteins at molecular, physiological and behavioral levels. In the present work we use the unique properties of magnetic resonance imaging (MRI) to probe the effects of altered dopaminergic dynamics on meso-scale neuronal circuitry and overall brain morphology, since changes at these levels of organization might help to account for some of the extensive pharmacological and behavioral differences observed in dopamine transporter (DAT) KO mice. Despite the smaller size of these animals, voxel-wise statistical comparison of high resolution structural MR images indicated little morphological change as a consequence of DAT KO. Likewise, proton magnetic resonance spectra recorded in the striatum indicated no significant changes in detectable metabolite concentrations between DAT KO and wild-type (WT) mice. In contrast, alterations in the circuitry from the prefrontal cortex to the mesocortical limbic system, an important brain component intimately tied to function of mesolimbic/mesocortical dopamine reward pathways, were revealed by manganese-enhanced MRI (MEMRI). Analysis of co-registered MEMRI images taken over the 26 hours after introduction of Mn^2+^ into the prefrontal cortex indicated that DAT KO mice have a truncated Mn^2+^ distribution within this circuitry with little accumulation beyond the thalamus or contralateral to the injection site. By contrast, WT littermates exhibit Mn^2+^ transport into more posterior midbrain nuclei and contralateral mesolimbic structures at 26 hr post-injection. Thus, DAT KO mice appear, at this level of anatomic resolution, to have preserved cortico-striatal-thalamic connectivity but diminished robustness of reward-modulating circuitry distal to the thalamus. This is in contradistinction to the state of this circuitry in serotonin transporter KO mice where we observed more robust connectivity in more posterior brain regions using methods identical to those employed here.

## Introduction

The dopamine transporter (DAT, SLC6A3) acts to terminate dopaminergic neurotransmission through reuptake of dopamine from the synaptic cleft into presynaptic neurons. Dopamine is a key neurotransmitter that can influence cognition, emotion, and movement; and many drugs exert their psychotropic effects via DAT [1], [2], [3], [4], [5]. In particular, dopamine plays an important role in the development and maintenance of addiction [6], [7] where much study has been devoted to its role in reward circuitry associated with the mesolimbic and mesocortical pathways [8], [9], [10], [11]. Dopaminergic neurons originate in the ventral tegmental area (VTA) and substantia nigra compacta (SNc), and projections to areas including the prefrontal cortex [12], integrate reward circuitry with executive functions mediated by the frontal cortex. The mesocortical and mesolimbic projections are part of the brain “reward circuit,” and are direct targets of psychostimulant drugs of abuse. This circuitry is also implicated in mental illnesses that include schizophrenia, major depressive disorder, and attention-deficit hyperactivity disorder [13], [14], [15]. Interactions among these, and other, structures are complex, with numerous opportunities for feedback involving a variety of connections and neuronal types (GABAergic, glutamatergic, dopaminergic, serotonergic, cholinergic, etc.) [16], [17], [18]. Mouse models with specific genetic modifications in the components of these pathways allow us to probe the ramifications of well-defined alterations with an eye toward parsing endophenotypes of pathological conditions and behaviors. Studies of mice with genetic manipulations of DAT [5], [19], [20], [21] and dopamine (DA) receptors [22], [23], [24], [25] have provided a wealth of information about the cellular, pharmacological, physiological and behavioral consequences of these manipulations.

In this work we link the ends of the molecular-to-behavioral spectrum using a panel of magnetic resonance imaging methods to investigate ramifications of DAT KO on mesoscopic scale neuronal circuitry and overall brain morphology in the mouse. By injecting tracer into the prefrontal cortex, we focus on the limbic cortical-ventral striatopallidal circuitry that has been implicated in a number of psychiatric disorders that are thought to involve changes in reward and executive functions mediated by the prefrontal cortex (PFC), including addiction [26], [27], [28], [29], [30], [31], [32]. This work parallels our previous examination of the serotonin transporter (SERT) KO mouse where significant differences in the reward circuitry were observed between SERT KO and WT mice [33]. In particular, we observed more robust connectivity between the PFC and posterior structures in SERT KO mice, using methods identical to those used in the present study.

Magnetic resonance spectroscopy (MRS), manganese enhanced MRI (MEMRI), and diffusion tensor imaging (DTI) data were obtained in DAT KO and WT mice using protocols similar to previous studies with SERT KO mice [33]. Magnetic resonance spectroscopy (MRS) of the striatum provides information about levels of several small molecules (*e.g.* glutamate, glutamine, lactate, N-acetyl-aspartate, choline, myo-inositol, taurine) that are present in the brain at millimolar or greater concentrations and can indicate disease states [34], [35], [36], [37]. MEMRI following stereotaxic injection of nanoliter amounts of Mn^2+^ into the PFC reveals active neuronal connections that emanate from the PFC. Computational co-registration of all the 3D images allows the determination of statistical parametric maps (SPM) that identify changes on a voxel-wise basis without the need to define regions of interest. These results thus provide a comprehensive unbiased approach to identification of the sites to which Mn^2+^ is carried from the PFC injection site. SPM analysis of intensity versus time provides information about how Mn^2+^ accumulation propagates from the injection site. Tensor based morphometry [38], [39] provides information about local size and shape. MRI makes possible 3D views of whole brain at 100 micron resolution, thus providing quantitative mesoscopic information about how deletion of DAT modulates metabolite levels, structure, and neuronal circuitry of executive and reward systems that are relevant to addiction.

## Results

### Injection site location and condition

**Figure 1:**
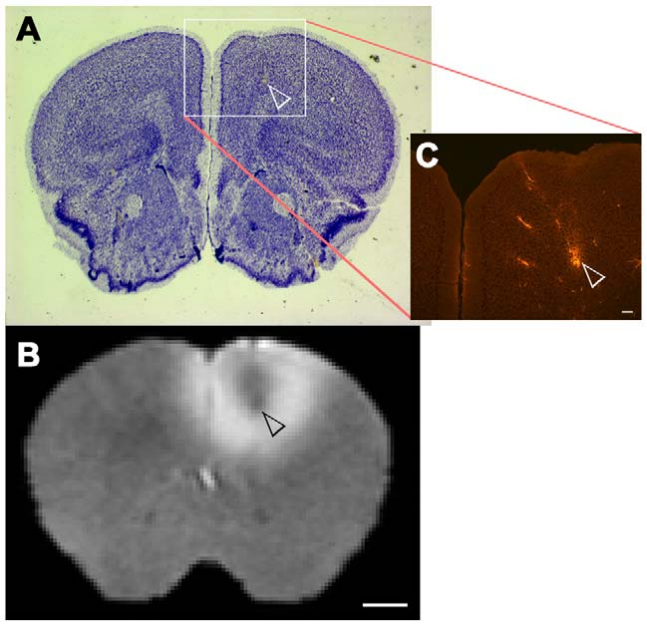
Histological examination revealed minimal damage at the injection site in the prefrontal cortex. A) Nissl-thionine stained section through the injection site; B) MRI slice from same animal as (A) in same location; C) Injection site, boxed region in (A) identified by imaging the same section shown in (A) for rhodamine-dextran fluorescence. The dextran fluorescence is easily visible against the background fluorescence from the Nissl stain. Scale bar for A and B  = 1 mm. Scale bar for C  = 100 µm.

Inspection of MR images recorded an hour after injection revealed that the center of each injection site fell within a 0.2 mm radius for all 16 animals. The average injection site (± standard deviation) was: x (lateral to the midline) +0.94±0.07 mm; y (anterior-posterior with reference to Bregma) +1.04±0.14 mm; z (dorsal-ventral from the brain surface) −1.29±0.21 mm. Histological examination of the injection site revealed minimal damage due to the injection procedure (Figure 1), and confirmed that the appropriate location was injected. Histology also confirmed that the co-injected retrograde tracer rhodamine-dextran was observed in the expected locations, such as the amygdale, providing an additional confirmation that the injections were correctly placed [40], [41] (Supplemental information Figure S1).

### Metabolite concentrations

We determined concentrations of metabolites relative to creatine plus phosphocreatine, which is assumed not to vary among mice or between the two genotypes, by probing the striatum of both genotype groups. All spectra were recorded from a 2 mm^3^ volume centered in the striatum. Relative concentrations for each metabolite are shown in Table 1. Student's t-test indicated that none of the metabolite levels were statistically different in the DAT KO vs WT littermates (p>0.05 in each case).

**Table 1:** Relative metabolite concentrations in striatum of DAT KO and WT mice are similar.

	Metabolite Ratio	
	DAT KO	WT
Choline	0.30±0.08	0.34±0.15
GABA	0.41±0.29	0.5±0.31
Glutamate	2.03±0.79	1.93±1.1
Glutamine	0.14±0.13	0.17±0.15
Lactate	0.54±0.36	0.57±0.39
Myo-inositol	0.86±0.32	0.83±0.30
N-Acetyl Aspartate	1.0±0.68	1.16±0.6
Taurine	0.37±0.31	0.45±0.25

Summary of results from MRS showing metabolite concentrations relative to creatine plus phosphocreatine.

### Voxel-wise Analysis of Morphometry and Mn^2+^ Accumulation

Image alignment allows voxel-wise comparisons in both human and small animal experiments [33], [42], [43], [44], [45], [46], [47]. Three separate statistical analyses were performed. The first, to gauge the extent of anatomical morphometric alterations resulting from the DAT KO, was done using tensor based morphometry (TBM) and high contrast data sets. The second gauged the impact of DAT KO on tissue structure manifest in altered water diffusional characteristics. For this analysis, we performed image alignment and statistical parametric mapping of images of a) fractional anisotropy (FA) and b) trace of the diffusion tensor (TrD) [48]. Thirdly, to follow the movement of Mn^2+^-induced hyperintensity in the MRI data sets, we performed image alignment and statistical parametric mapping to compare how DAT KO and WT mice differed in the time dependence of Mn^2+^ accumulation at locations distant from the injection site.

For the tensor based morphometry analysis, the structural and isotropic diffusion weighted (iDWI) data sets from DAT KO and WT mice that were obtained prior to any injections provided input. Very few voxels displayed deformation tensor indices that differed between DAT KO and WT mice. At a p<0.001 level of significance no voxels were significantly different in the logmat SPM. Only 0.8% of voxels reached this level of significance in the det SPM.

**Figure 2:**
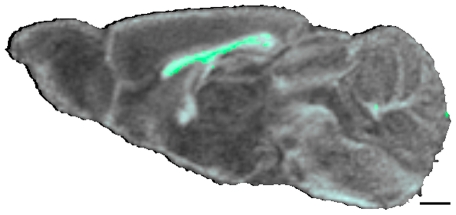
Fractional anisotropy differences between DAT KO and WT. A para-sagittal slice through the average of all WT FA data sets (grayscale background) is shown. Voxels that differ significantly between the DAT KO and WT brain scans at P<0.005 are indicated in green. Scale bar  = 500 µm.

Fractional anisotropy and trace of the diffusion tensor are two rotationally-invariant indices derived from the diffusion tensor that provide information about underlying tissue characteristics [49], [50], [51], [52], [53], [54], [55], [56]. Fractional anisotropy is a scalar measure of the diffusion anisotropy (*i.e.* the propensity of water to diffuse along, rather than perpendicular to, a nerve bundle). TrD is a measure of the orientation-independent mean diffusivity of water in the tissue. Figure 2 shows a sagittal slice through the average of all WT FA data sets (gray background). Green voxels differ significantly between the DAT KO and WT brain scans. FA measurements in the corpus callosum, adjacent external capsule and a small part of cerebellar commissure differed between the two genotypes at p<0.005. Corpus callosum FA was ∼24% less in DAT KO mice compared with WT mice. The cerebellar commissure FA was ∼35% less in DAT KO mice. No other regions were significantly different. No differences were found in the TrD maps of DAT KO compared with WT at p<0.005.

**Figure 3:**
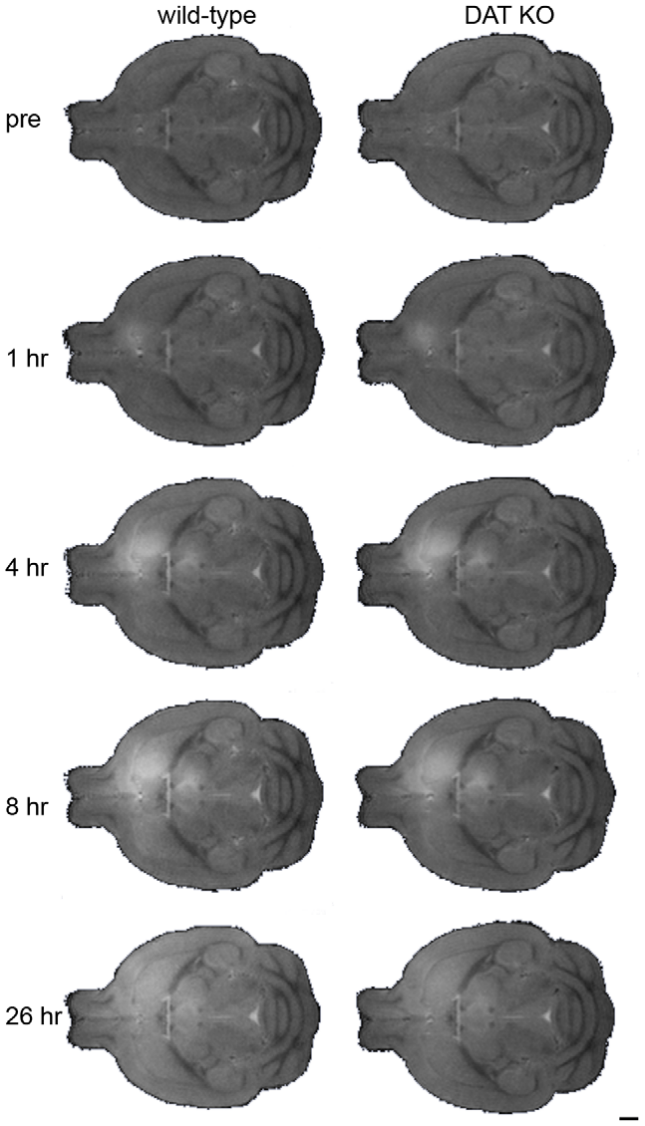
The minimum deformation template (MDT) images preserve the fidelity of the original images. The minimum deformation target (MDT) demonstrates preservation of the fidelity of the original images. Note the clarity of anatomical structures. The center of the injection site in the PFC is 1 mm above these transverse sections, which are at 2.8 mm below the brain surface. These sections illustrate the result of aligning all the DAT KO and WT *in vivo* MR images as described in Materials and Methods. Hyperintense regions indicate the location of Mn^2+^ as it moves away from the injection site. Scale bar  = 1 mm.

**Figure 4:**
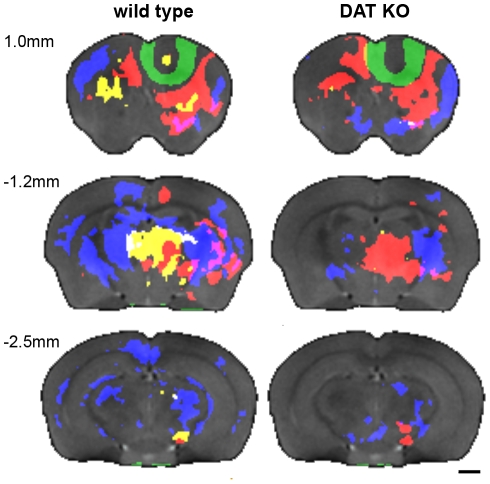
Coronal sections of statistical parametric maps show the progression of Mn^2+^ accumulation over time. Representative coronal sections through the statistical parametric maps show the progression of Mn^2+^ accumulation over time. Gray background is pre-injection MDT, while the green (1 hr > pre-injection), red (4 hr >1 hr), yellow (8 hr >4 hr) and blue (26 hr >8 hr) overlays indicate areas with increased intensity (P<0.00025) compared to the preceding time point. Video in Supplemental Data shows the complete image data sets in for each genotype (Video S1). Slice locations are indicated relative to Bregma. Scale bars  = 1 mm.

**Figure 5:**
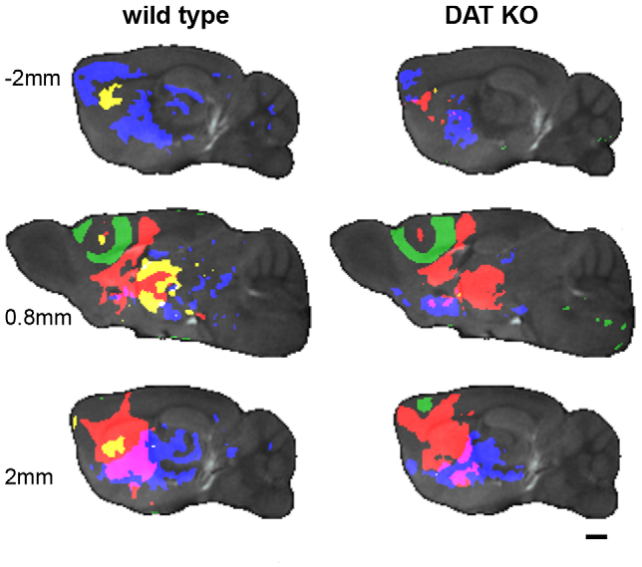
Sagittal sections of statistical parametric maps show the progression of Mn^2+^ accumulation over time. Representative sagittal sections through the statistical parametric maps show the progression of Mn^2+^ accumulation over time. Gray background is pre-injection MDT, while the green (1 hr > pre-injection), red (4 hr >1 hr), yellow (8 hr >4 hr) and blue (26 hr >8 hr) overlays indicate areas with increased intensity (P<0.00025) compared to the preceding time point. Video in Supplemental Data shows the complete image data set for each genotype (Video S2). Slice locations are indicated relative to the midline. Scale bars  = 1 mm.

**Figure 6:**
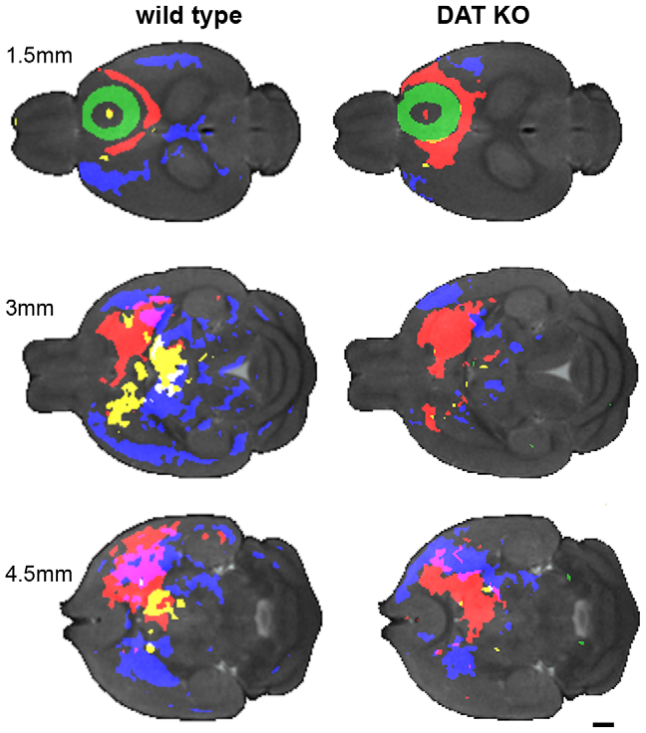
Transverse sections of statistical parametric maps show the progression of Mn^2+^ accumulation over time. Representative transverse sections through the statistical parametric maps show the progression of Mn^2+^ accumulation over time. Gray background is pre-injection MDT, while the green (1 hr > pre-injection), red (4 hr >1 hr), yellow (8 hr >4 hr) and blue (26 hr >8 hr) overlays indicate areas with increased intensity (P<0.00025) compared to the preceding time point. Videos in Supplemental Data show the complete image data sets for each genotype (Video S3). Slice locations are indicated relative to the brain. Scale bars  = 1 mm.

Figure 3 illustrates the results of two procedures: a) aligning all the DAT KO and WT i*n vivo* MR images then b) calculating the minimum deformation target (MDT) at each time-point, which provides an unbiased average. The MDT images preserve the fidelity of the original images. Anatomical structures are clear and distinct; ventricles, corpus callosum, hippocampus, internal and external capsule are all easily identifiable. To provide an unbiased assessment of the whole brain, we performed statistical parametric mapping to determine the spatial extent of Mn^2+^ accumulation, detected by Mn^+2^-induced signal intensity increase, as a function of time after injection. Color-coded volumes denote significant (p<0.00025, uncorrected) increases in intensity from one time point to the next. Representative slices from the 3 dimensional data sets are shown in Figures 4, 5 and 6. Videos showing the entire 3D data sets are available as supplementary information (Video S1, S2, S3 and S4). For clarity, WT and DAT KO data are shown separately. The earliest time period comparison is shown in green (1 hr > pre-injection), the 4 hr >1 hr comparison is shown in red, the 8 hr >4 hr comparison is shown in yellow, and the 26 hr >8 hr comparison is shown in blue for both DAT KO and WT mice.

The injection site is highlighted (green). This denotes the significantly more intense signal induced by injecting Mn^2+^ into the prefrontal cortex (Figures 4, 5 and 6). As the green areas (1 hr > pre) show in the coronal (Figure 4), sagittal (Figure 5), and transverse (Figure 6) views, Mn^2+^ induced hyperintensities spread outward in an essentially isotropic manner immediately after the injection to provide a spheroid of hyperintensity in the MR image. This spreading is likely due to diffusion of the Mn^2+^ ion. In DAT KO mice, hyperintense volume elements are also seen along the midline immediately below the 4^th^ ventricle (4V). To check that the intensity increase near 4V was not due to a single outlier, we repeated the Student's *t*-test parametric map analysis 8 times, each time dropping a different single sample from the analysis. The intensity increase near 4V was observed in all the comparisons.

Student's *t*-test comparisons of the 4 hr post-injection image datasets with those recorded at 1 hour reveal widespread changes (Figures 4, 5 and 6, red areas, 4 hr >1 hr). Ventral to the injection sites, DAT KO mice display ipsilateral hyperintensity increases that expand into the ipsilateral motor cortex (M1 and M2), nucleus accumbens, globus pallidus, much of the striatum, lateral amygdala, middle anterior thalamus, and small areas farther posterior to the substantia nigra (see Table 2). Dorsal and ipsilateral to the injection site, the DAT KO mice showed increased hyperintensity in parts of the cingulate cortex, anterior striatum, and near the midline in the thalamus. WT littermates showed a similar pattern to the DAT KO mice with one exception: some hyperintensity increase was seen in the subiculum at more than 3 mm from the midline ipsilateral to the injection site.

**Table 2:** Anatomical features highlighted by MEMRI as a function of time after PFC stereotaxic injection.

Anatomical feature	1 hr	4 hr	8 hr	26 hr	SERT 25 hr
PFC: prefrontal cortex	KO & WT				
Cortex (S, M, Cg, M, RS)		KO & WT		KO (S only) & ***WT***	
CP: caudate putamen		KO & WT	WT_IC_	KO_IC_ & WT_IC_	WT
VP: ventral pallidum		KO & WT		KO_ic_ & WT_IC_	
ACB: nucleus accumbens		KO & WT		KO_ic_ & WT_IC_	WT_ic_
GP: globus pallidus		KO & WT		KO_IC_ & WT_IC_	KO & WT_ic_
AMG: amygdala		KO & WT			
TH: thalamus		KO & WT	WT_IC_	KO_ic_ & WT_ic_	
HY: hypothalamus LH		KO & WT		***WT_IC_***	KO
SI: substantia innominata					WT_ic_
SNr: substantia nigra		WT	WT	KO & **WT**	KO_ic_
MG: medial geniculate nu				***WT***	
VTA: ventral tegmental area					KO
RN: red nucleus					KO
PRN: pontine reticular nucleus					KO
MEnt: medial entorhinal cortex				***WT_IC_***	
DpMe: deep mesencephalic nu				***WT***	
subiculum		**WT**		***WT***	
PAG: periaqueductal gray				***WT_IC_***	
midline near 4V	**KO**				

A synopsis of anatomical features highlighted by Mn^2+^ MRI as a function of time after injection into the prefrontal cortex. At 26 hrs these anterior cortical areas are highlighted in the WT mouse: S, M, Cg, and RS. For comparison, the rightmost column (SERT 25 hr) shows features highlighted in our previous SERT KO study at 25 hr after Mn^2+^ injection into the prefrontal cortex [33]. Anatomical features were identified by comparing images like those shown in Figure 4 with standard mouse brain atlases ([119] and the Allen Brain Atlas [120]). KO: DAT KO; WT: WT control; no subscript: ipsilateral; IC subscript: ipsilateral & contralateral.

Comparison of the 8 hr images with the 4 hr images shows little change for the DAT KO with minor increases in the ipsilateral accumbens and contralateral striatum. In contrast, the WT mice displayed hyperintensity increases in both the ipsilateral and contralateral striatum and thalamus, and ipsilateral substantia nigra (Figures 4, 5 and 6, yellow areas).

Between 8 hr and 26 hr, hyperintensity increases in the DAT KO mice were seen both ipsilateral and contralateral to the injection site in the striatum, accumbens, globus pallidus, and amygdala. The substantia nigra and mid thalamus had increased hyperintensities mostly ipsilateral with only small increases contralateral to the injection site (Figures 4, 5 and 6, blue areas). In the WT mice, a greater number of areas showed increases: bilaterally in the striatum, globus pallidus; parts of the somatosensory and retrosplenial granular cortices; portions of the hypothalamus and much of the ventral thalamus. Substantial enhancement was seen ipsilateral to the injection site in the substantia nigra while contralateral enhancement was seen in the cingulate cortex.

## Discussion

In this study we have used a panel of magnetic resonance methods, as well as histological confirmation, to investigate changes brought about by lifetime genetic deletion of the dopamine transporter. This study parallels an earlier examination of the serotonin transporter knockout mouse [33]. Histology confirmed that stereotaxic injection of 3 nanomoles of Mn^2+^ with fluorescent tracers was minimally invasive and placed in the desired forebrain location. There was little evidence of toxicity, in accord with work of Canals and coworkers who found that higher amounts were required to induce significant astrogliosis and neuronal cell death (8 and 16 nanomoles of Mn^2+^, respectively). [57].

Neither metabolite concentrations nor anatomical differences were found between DAT KO mice and their WT littermates. MRS detectable metabolite concentrations in the striatum were at similar levels in WT and DAT KO mice. There was thus no evidence that these animals differ from wildtype mice in neuronal viability (indicated by NAA), excitatory and inhibitory system integrity (indicated by Glu and GABA), membrane turnover (indicated by Cho), or glial volume (indicated by Tau) [35], [58], [59], [60], [61], [62], [63], [64]. Similarly, no differences were observed for these measures between WT and SERT KO mice in our previous study [33].

Tensor based morphometry compared DAT KO and WT brains, based on *in vivo* pre-injection structural MRI and *ex vivo* iDWI data sets. These assessments also revealed few statistically significant differences between genotypes. Although DAT KO mice are known to have somewhat smaller brains than WT mice (consistent with overall smaller body size [65]) within the constraints of resolution (100 µm) and contrast of the MRI data used, the TBM analysis indicated little or no gross anatomical differences between WT and DAT KO mice. Thus, there were no differences in the size or shape of anatomical structures, no missing structures, no cerebellar or hippocampal dismorphisms. Using a combination of manually segmented high resolution MRI and stereological analysis, Cyr and coworkers [66] observed that the anterior striatum (but not the posterior striatum) of DAT KO mice was 9% smaller as compared to wild-type mice. Further, using histological analysis they found an 18% decrease in the number of neurons in the anterior striatum of DAT KO mice as compared to wild-type mice, while the number of glial cells was unchanged. The higher spatial resolution (43 µm) and somewhat better contrast to noise ratio obtained with the techniques used in the previous work make it more sensitive to small morphological changes than the current study.

The fractional anisotropy and the trace of the water diffusion tensor have been used to gain insight into changes in white matter tracts and other ordered brain structures [47], [67], [68]. To further characterize structural differences between DAT KO and WT mice we compared FA and TrD images of the two genotypes using statistical parametric maps. No statistically significant differences were seen in TrD maps (p<0.005). FA in the corpus callosum and adjacent external capsule was ∼24% less in the DAT KO mice compared to WT mice (see Figure 2) and a small portion of the cerebellar commissure had FA ∼35% less in the DAT KO. Although these are relatively small changes, lower fractional anisotropy could be a result of dysmyelination and/or less order in the neuronal structures crossing the midline in the DAT KO compared to WT mice that might indicate altered communication between the two hemispheres.

**Figure 7:**
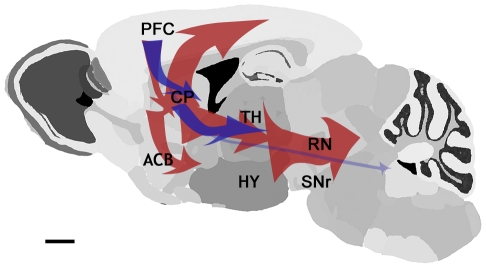
Schematic representation shows PFC associated circuits are more robust in WT compared to DAT KO mice. Major circuits delineated by MEMRI are shown. Red (WT) and blue (DAT KO) arrows in the schematic diagram indicate Mn^2+^ transport in the two genotypes. Detailed anatomy and timing of Mn^2+^ transport are shown in Table 2.

MEMRI revealed major differences in the pattern of connectivity between the prefrontal cortex and distal brain regions between the DAT KO mice and their WT littermates. The complex statistical parametric maps can be assembled into a simplified diagram of the anatomical locations occupied as Mn^2+^ induced- hyperintensities move from the injection site to more distant locations in the brain (Figure 7). This schematic diagram and list of structures in Table 2 reveal that anatomical features highlighted by Mn^2+^ tracing are somewhat different in the DAT KO mice as compared to WT littermates. Statistical parametric mapping in the WT mice shows Mn^2+^ tracing to the striatum, thalamus, substantia nigra, and subiculum by 4–8 hr post injection. By 26 hr post-injection, WT mice showed widespread MRI intensity increases that encompassed the contralateral thalamus, several cortical areas (especially the somatosensory cortex), and more basal anterior structures. Thus, WT mice display Mn^2+^ accumulation reflecting the same connectivity along the cortico-mesolimbic pathway as has been assessed in classical tracing work [69], [70], [71], [72]. In contrast to the connections from the PFC seen in WT mice, in the DAT KO mice Mn^2+^ movement was truncated at 26 hr post injection. In the DAT KO mice at 26 hr post-injection the hyperintensity distribution includes a substantially smaller subset of the WT distribution and Mn^2+^ accumulation failed to reach such posterior structures as medial entorhinal cortex, deep mesencephalic nuclei, and subiculum. Thus, deletion of the DAT gene produces alterations in mesocortical circuitry that originate from the PFC.

The time-course and spatial extent of the circuitry traced out using MEMRI in the WT littermates of DAT KO mice was substantially different than that observed for the WT littermates of SERT KO mice reported in our previous study, although the reasons for these differences are not clear [33]. The studies of these two strains use the same injection site and procedure. The genetic background is similar between the two strains, being comprised of C57BL/6J and 129Sv alleles. While we have not observed differences between C57BL/6J and 129X1/SvJ mice using MEMRI (Jacobs, unpublished results); it is likely that different combinations of C57BL/6J and 129Sv alleles have become fixed in each strain. The differences that we identify here do emphasize the necessity for littermate comparisons in transgenic studies to control for the effects of genetic background.

As in all studies employing neuronal tracers, interpretation of the results depends on the properties of the tracer. Mn^2+^ is primarily an anterograde trans-synaptic tracer that is transported via fast axonal transport and accumulates intracellularly to levels sufficient to induce increased intensity in T1-weighted MR images at locations distant from the injection site [73], [74], [75]. Mn^2+^ is an activity dependent tracer that is taken up by active neurons, probably via voltage and/or ligand gated Ca^2+^ channels [76], [77], [78]. There is no known specificity of Mn^2+^ for any particular type of neuron. However, although all active neurons at the site of injection may have taken up the tracer, the initial projections observed in this work are likely to arise from the pyramidal projection neurons of the prefrontal cortex [79].

Studies of DAT KO mice have used a wide variety of behavioral, pharmacological, physiological, and ultrastructural measures to suggest that this knockout induces significant adaptive changes at the synaptic, cellular, and molecular levels. These data include region-specific changes in various dopamine receptor densities and altered responses in other neurotransmitter systems [80], [81], [82], [83], [84], [85]. Thus, it is reasonable to assume that differences between DAT KO and WT mice observed in this study are due to local differences in neuronal circuitry and/or activity at both the injection site and at distal synapses. Alterations in transport or intracellular accumulation seem less likely to explain the observations made in the present report.

At the earliest time-point in our series (1 hr post-injection), Mn^2+^ accumulation is seen in the DAT KO mice on the midline bordering the base of the fourth ventricle. Such accumulation could be either a consequence of migration around the brain of Mn^2+^ leaked at the brain surface during the injection procedure or fast axonal transport through the brain parenchyma. Timing of passage around or through the cerebrum are both consistent with appearance of Mn^2+^ at this location an hour after its introduction. Within half hour of introduction into the cisterna magna of the rat, india ink tracer distributes as far forward as the olfactory bulbs and along paravascular pathways of the middle cerebral artery and its branches on the superior surface of the brain [86]. Estimates of the rate of fast axonal transport range from 2 to 16 mm/hr [87], [88], [89], [90]. The minimum distance between the injection site and 4V is 6.5 mm, while the distance measured from the injection site through the striatum and then onto 4V is 7.5 mm. Thus, even through the longer more anatomically appropriate pathway, the rate of transport observed here is within the range of previously observed rates. Rapid Mn^2+^ accumulation in areas near 4V may indicate active connections from the prefrontal cortex to periventricular areas below 4V in the DAT KO mouse that are not present, at least to the same extent, in their WT littermates. Limited resolution and contrast in the MR images and parametric maps coupled with the existence of many small nuclei in this area preclude definitive identification of the specific structures involved.

Over the course of 4 hours, Mn^2+^ is transported from the prefrontal cortex to most of the expected areas in both WT and KO mice: caudate putamen (CP), globus pallidus (GP), nucleus accumbens (NAc), thalamus (TH), and substantia nigra (SNr). These areas represent a large subset of the known connections between the neocortex and the basal ganglia of the rodent brain [69], [71], [72]. There was little change in Mn^2+^ accumulation between 4 hr and 8 hr in the DAT KO (note almost no yellow areas in DAT KO, Figures 4, 5 and 6). Whereas, in the WT there are substantial increases, generally ipsilateral, in the striatum, thalamus, and substantia nigra. All these structures had already seen Mn^2+^ accumulation at the earlier 4 hr time point in both genotypes. That Mn^2+^ continues to accumulate in more posterior anatomy in the WT and not the KO is further indication of more robust connectivity in the WT as compared with the KO.

In WT mice extensive differences accrued between 8 hr and 26 hr, while only incremental changes were observed in DAT KO mice. In DAT KO mice Mn^2+^ accumulation was restricted to structures observed at 4 hr post-injection with expansion within the striatum and thalamus. Whereas, in WT mice large areas both ipsilateral and contralateral to the injection site showed hyperintensity increases at the later time points. These included areas highlighted at 8 hr (*e.g.* striatum and thalamus – see yellow colored areas in Figures 4, 5 and 6), as well as portions of the cortex (especially somatosensory and retrospenial cortices) and midbrain regions extending more posterior to the substantia nigra, medial geniculate nuclei, medial entorhinal cortex and subiculum. The lack of widespread Mn^2+^-induced hyperintensity in DAT KOs observed 26 hr post-injection indicates a similarly diminished general communication among distant brain regions in these mice compared to WT littermates. Less robust connectivity to more posterior areas would be a natural consequence of cortical ‘hypofrontality’ and/or a consequence of poorer communication from the striatum. Interestingly, chronic exposure to any of several drugs of abuse is associated with reduced activity in the frontal cortex [7], [11], [91], [92]. The previously observed decrease in the number of anterior striatal neurons in the DAT KO mouse [66] would be expected to diminish connectivity to structures functionally downstream of the striatum, as was observed here. As the PFC and striatum use, or receive, projections from a number of signaling systems (dopaminergic, glutamatergic, gabaergic, serotonergic, noradrenergic, etc.) [72], [93], [94], [95], [96], [97], widespread differences in Mn^2+^ accumulation at 26 hr between DAT KO and WT mice are likely to represent of indirect consequences of alterations in dopaminergic signaling induced directly by deletion of DAT.

By 26 hr, Mn^2+^ had sufficient time to cross many synapses and to be transported throughout the mouse brain [98]. Thus, the Mn^2+^ induced hyperintensity at 26 hr may have been a reflection of local activity as well as connectivity, complimenting PET ^18^FDG uptake studies. Recent PET ^18^FDG uptake studies in DAT KO mice indicate differential brain metabolism levels in KO versus WT mice in both the resting state and after acute cocaine exposure [92]. DAT KO mice have marginally higher baseline ^18^FDG uptake in the thalamus and cerebellum compared to WT mice, while all other areas examined had similar levels of uptake. Acute cocaine exposure decreased ^18^FDG uptake in the olfactory bulb, motor cortex, striatum, hippocampus, TH, and cerebellum in WT mice, while in DAT KO mice only the TH showed a significant decrease in glucose metabolism. In comparison, Mn^2+^ accumulation from 8 hr to 26 hr observed in WT mice was substantially more widespread (*e.g.* cortical regions, ipsilateral and contralateral TH, and periaqueductal gray areas) than that observed in DAT KO mice. When basal activity differences are conflated with connectivity differences, as they are in MEMRI, it is not surprising that somewhat different patterns emerge in the MEMRI brain scans compared to techniques that measure basal metabolism only, such as ^18^FDG uptake in PET scans. Nonetheless, these imaging results, taken together, indicate that the DAT KO brain is both qualitatively and quantitatively different than the corresponding WT brain, with substantial effects on the reward pathways that may involve several of the monoamine systems.

The DAT KO mouse has been suggested to constitute an animal model of several disorders thought to involve hyperdopaminergic function, including schizophrenia [2], [99], which would be consistent with a “hypofrontal” phenotype. To some extent this is a consequence of altered prefrontal/striatal dopamine function that arises from the DAT KO. Although basal extracellular dopamine levels in the striatum are greatly enhanced in DAT KO mice compared to WT mice, this is not observed in the PFC [100]. This circumstance results in a shift in the balance of dopaminergic activity, as well as, it would seem, the overall balance of activity within other portions of the reward circuitry. Thus, there are substantial differences in Fos-like immunoreactivity between DAT KO and WT mice after psychostimulant administration. DAT KO mice show substantially reduced activation of frontal areas [101] as well as a “hypofrontal” phenotype observable in PET imaging [92]. Some behavioral changes are also consistent with this overall picture. For instance, DAT KO mice have deficits in pre-pulse inhibition of startle that can be ameliorated by dopamine D2 receptor antagonists [102]. How valid a model the DAT KO mouse is as an animal model of schizophrenia remains to be seen, but, in any case, several other findings support the overall picture described above. The most overt consequence of the DAT KO is locomotor hyperactivity [103], [104] that has been described as constituting an animal model of attention deficit hyperactivity disorder [99]. Interestingly, both hyperactivity and impairments of pre-pulse inhibition of startle in DAT KO mice can be reversed by stimulant drugs such as cocaine, amphetamine and methylphenidate [105], [106]. Indeed, in each case the actions of the psychostimulants were in the opposite direction to that observed in WT mice: hyperactivity and attentional impairments in WT mice, and reduced hyperactivity and amelioration of attentional deficits in DAT KO mice. Importantly, it was shown that the effects of these drugs on pre-pulse inhibition in DAT KO mice were actually mediated by NET blockade [106], which presumably acts selectively in the prefrontal cortex (but not the striatum) to elevate dopamine levels and restore the balance of cortical and striatal dopamine function. Overall, this literature is consistent with the present results which indicate reduced fronto-cortical activity in DAT KO mice, indicating that many DAT KO phenotypes are not simply the result of increased DA function, but of the consequent changes in activity within the overall circuitry of which the dopaminergic projections are just one part.

In this study we employed a panel of magnetic resonance methods to probe the effects of lifelong deletion of DAT in a mouse model system. As detected by magnetic resonance methods, no significant changes in metabolite levels or morphology were observed in DAT KO mice. In contrast, MEMRI revealed that DAT KO mice had extensive alterations in the executive portions of reward circuitry that are intimately involved in addiction, as well as other psychiatric conditions. Connections originating in the prefrontal cortex were significantly truncated in the DAT KO mice. The changes we observed are consistent with cortical ‘hypofrontality’, described in behavioral, pharmacological and neurochemical studies of DAT KO mice. In contrast to the present results, our previous study of the SERT KO mouse revealed a generally more robust cortico-limbic circuit compared to WT mice. These studies indicate that a panel of MR imaging methods combined with statistical parametric mapping analyses is a powerful tool for endophenotyping the pathological manifestations of genetic alterations in executive/reward circuitry.

## Materials and Methods

### Animals

The line of DAT KO mice used in these experiments has been described previously [104]. Eight DAT KO mice and eight WT littermates were used in this study. Mice were mixed male and female subjects between the ages of 18 and 24 weeks.

### Ethics Statement

All protocols involving animals in this work were approved by the Institutional Animal Care and Use Committee of the California Institute of Technology.

### Stereotaxic Injections

The stereotaxic injection procedure was similar to that employed by Bearer et al [107]. Mice were anesthetized with ketamine/xylazine (7.5 mg ketamine plus 5 mg xylazine per kg, i.p.) and placed in a stereotaxic frame (Kopf Instruments, Tujunga, CA). 5 nl of 600 mM MnCl_2_ with 0.5 mg/ml rhodamine dextran-amine (3k) (Molecular Probes/Invitrogen, Eugene, OR) was injected unilaterally into the right prefrontal cortex (coordinates *x* –0.8 mm (from the midline), *y* +1.9 mm (from Bregma), *z* 1.8 mm (from the dorsal surface of the brain) [108]) over 5 minutes using a quartz micropipette (1 mm OD quartz capillary pulled to approximately 20 micron OD tip) guided by computer-assisted stereotaxic injector (myNeuroLab.com, Richmond, IL). The animal was immediately placed in the MR scanner under 0.8% isoflurane anesthetic.

### Preparation for *ex vivo* imaging

Within 10 days after *in vivo* imaging, animals were sacrificed and brains fixed via transcardiac perfusion with 4% paraformaldehyde (PFA) as previously described [47]. After overnight rocking in 4% PFA at 4°C the mouse head was cleaned of skin, lower jaw, ears and cartilaginous nose tip and then rocked in 50 ml 0.01% sodium azide in PBS for 7 days at 4°C. The head was then transferred to a 5 mM solution of gadoteridol (Prohance®, Bracco Diagnostics Inc, Princeton NJ) and 0.01% sodium azide in PBS and rocked for 7 days at 4°C prior to MR imaging.

### Magnetic Resonance Imaging and spectroscopy

Each animal was scanned before the stereotaxic injection; and beginning at 0∶54±0∶13, 4∶23±0∶09, 8∶13±0∶06, and 25∶45±0∶48 hours post injection. Times are averages over all animals ± standard deviation. We use the midpoint of each 40 minute scan as the “scan time” and for convenience call these the 1 hr, 4 hr, 8 hr, and 26 hr time points. An 11.7 T 89 mm vertical bore Bruker BioSpin Avance DRX500 scanner (Bruker BioSpin Inc, Billerica, MA) equipped with a Micro2.5 gradient system was used to acquire all mouse brain images and spectroscopic data with a 35 mm linear birdcage radio frequency (RF) coil. For *in vivo* imaging the animal's head was secured in a Teflon stereotaxic unit within the RF coil to minimize movement and to aid in reproducible placement. Temperature and respiration were continuously monitored during data acquisition and remained within normal ranges. We employed a 3D RARE imaging sequence [109] with RARE factor of 4, 4 averages, TR/TE_eff_  = 250 ms/12 ms; matrix size of 160×128×78; FOV 16 mm ×12.8 mm ×7.8 mm; yielding 100 µm isotropic voxels with 40 minute scan time.

All *in vivo* mouse brain magnetic resonance spectroscopy (MRS) experiments were conducted using Point Resolved Spectroscopy (PRESS) [110] with a short echo time TE of 7.267 ms, recycle time of 2.3 seconds, a spectral width of 7 KHz, 4000 data points in each free induction decay signal (FID), and 128 averages. The sequence was preceded by a VAPOR water suppression module [111] interleaved with outer volume saturation. Optimized second order shimming was done with the FastMap routine [112] in a 5 mm cube centered in the striatum. The PRESS spectra were then recorded inside a 2 mm^3^ volume (8 µl) at the center of the volume used for shimming.

For *ex vivo* imaging, two intact heads were secured in a Teflon® holder and submerged in perfluoropolyether (Fomblin®, Solvay Solexis, Inc, Thorofare, NJ) within a 50 ml vial and imaged. The ambient bore temperature was maintained at 4°C by thermostatically controlled airflow. Diffusion weighted images were acquired using a conventional pulsed-gradient spin echo (PGSE) sequence (TR/TE  = 300 ms/11.9 ms, 256×150×120 matrix, 25.6 mm ×15 mm ×12 mm FOV, 100 µm isotropic voxel size, 1 average, δ = 3 ms, Δ = 5.2 ms, Gd = 1125 mT/m, nominal b-factor  = 3370 s/mm^2^). An optimized six point icosahedral encoding scheme [113] was used for diffusion weighted acquisitions with a single un-weighted reference image for a total imaging time of 14.5 hours.

### Histology

After DTI imaging, 5 brains were selected for histological examination, 3 DAT KO and 2 WT littermates. Prior to processing, each brain was released from the skull, post-fixed 1–3 days, and then sent to Neuroscience Associates (NSA, Knoxville, TN) for gelatin embedding and frozen sectioning as described [33]. All 5 brains were embedded in a single gelatin block and sectioned in register at 30 µm thickness. Each 12th section was stained for Nissl-Thionine and an adjacent section mounted in DAPI-containing anti-quench and mounted for fluorescence microscopy of the co-injected rhodamine dextran. Sections were imaged on a Zeiss V8 stereoscope equipped with an Axiocam running AxioVision 4.8, bright and dark-field illumination and three fluorescent filter cubes for DAPI, TRITC, and FITC on a Zeiss Axioscope Z1 with an MRM and HRC Axiocam running AxioVision 4.6 software and having a similar brightfield and fluorescence capabilities.

### Image Alignment and Statistical Parametric Mapping

Pre- and post-Mn^2+^ injection MR images were skull-stripped using the Brain Surface Extractor (BSE) within BrainSuite2 [114] to remove all non-brain material. Inaccuracies were corrected by manually editing the masks using BrainSuite 2. After skull-stripping, field inhomogeneities were corrected using the N3 method [115] and each was scaled to the mode of its intensity histogram [107], [116], [117]. A minimum deformation target (MDT) was produced from the pre-injection images as described [33], [118]. The pre-injection images were then warped into this MDT as described [33] and the post-injection images were linearly aligned (12 parameter model) to the pre-injection images using Alignlinear, followed by application of the polynomial warp field used to transform that sample's pre-injection image into the MDT. Final images were blurred with a 0.3 mm Gaussian kernel and a paired Student's t-test was used to determine which voxels increased in intensity when comparing one time point to the next. Similar processing was used to compare rotationally invariant indices derived from DTI datasets, except that an unpaired Student's t-test was used to compare DAT KO and WT mice. Parametric maps of voxels with statistically significant changes in intensity were created to display the results and to correlate increases with underlying anatomy [107]. Anatomy was determined with reference to Hoff et al. [119] and the Allen Brain Atlas [120] (http://www.brain-map.org/welcome.do).

### Tensor Based Morphometry

A deformation field analysis implemented by Thompson and coworkers [121] was used to analyze whether MRI scans of the DAT KO mice differed anatomically from WT mouse brain scans, as described [33]. Structural RARE and iDWI data were mapped to their respective MDTs, the deformation field needed to warp each image to the WT pre-injection structural or iDWI MDT determined, and p-value maps calculated noting statistical significance of differences in the deformation tensors of the two cohorts as measured by comparisons of the determinant of the deformation tensor and matrix logarithm of the determinant at each voxel [39], [48], [117], [122], [123].

### Determination of relative metabolite concentrations

For each mouse brain the spectrum of the relative amount of metabolites inside the experimental PRESS volume was quantified using the QUEST (quantitation based on quantum estimation) module [124] available inside the Java Magnetic Resonance User Interface (JMRUI) package [125]. Spin parameters (number of spins, chemical shifts, J-couplings) were obtained from Govindaraju [126]. Metabolite amounts obtained by the QUEST were normalized to creatine plus phosphocreatine.

### Diffusion tensor image construction

Reconstruction of the apparent diffusion-weighted images included spatial radial Gaussian filtering (0.25 voxel width) to smooth the co-registration cost-function and improve the SNR of all subsequent calculations. The apparent diffusion tensor was calculated conventionally by inversion of the encoding b-matrix. The b-matrix for each diffusion encoding was determined by numerical simulation of the pulse sequence k-space trajectory in order to account for gradient cross-terms [127]. Eigenvalues, eigenvectors, tensor trace and fractional anisotropy were calculated conventionally using built-in and custom Matlab functions (The Mathworks Inc., Natick MA). The six diffusion weighted images were averaged to generate a high SNR isotropic diffusion weighted image (iDWI). Diffusion tensor images (and associated images of eigenvalue, λ_i_; trace, TrD; and fractional anisotropy, FA) were placed into the same space for voxel-wise comparisons using the methods outlined above for *in vivo* MR images.

### Rendering

Visualization of the MR images and statistical parametric maps was performed with ResolveRT4 (Mercury Computer Systems, Inc., Hudson, NH) and MRIcro [128].

## Supporting Information

Figure S1 Rhodamine-dextran is transported to the amygdala in a wild type animal 16 days after injection in the PFC. The sections was mounted, stained with DAPI for nuclei and imaged for rhodamine and DAPI fluorescence. Alternate sections were Nissl stained. A) Rhodamine-dextran appears in the red channel in neurons of the amygdala and not in other areas in this part of the brain. The DAPI-stained nuclei are shown in blue. B) Nissl-stained section adjacent to the section shown in A) which overlaps the boxed area. These results demonstrate that the location of the injection site was appropriate for the introduction of tracer into the expected forebrain-midbrain pathway. The location of the rhodamine fluorescence is consistent with rhodamine-dextran having arrived via retrograde transport along the axons to the neuronal cell body. Scale bar  = 100 µm.
(3.23 MB TIF)

Video S1 Coronal sections through the statistical parametric maps show the progression of Mn2+ accumulation over time. Gray background is pre-injection MDA, while the colored overlays denote areas with increased intensity (P<0.00025) at 1 hr (green), 4 hr (red), 8 hr (yellow), and 24 hr (blue) compared to the preceding time point. Video shows consecutive sections in the coronal directions for the DAT knockout and wild-type cohorts. Slice locations are shown at the bottom right in millimeters with respect to Bregma. Scale bar  = 1 mm.
(7.36 MB MOV)

Video S2 Sagittal sections through time-series statistical parametric maps. Gray background is pre-injection MDA, while the colored overlays denote areas with increased intensity (P<0.00025) at 1 hr (green), 4 hr (red), 8 hr (yellow), and 24 hr (blue) compared to the preceding time point. Video shows consecutive sections in the sagittal directions for the DAT knockout and wild-type cohorts. Slice locations are shown at the bottom right in millimeters with respect to midline. Scale bar  = 1 mm.
(6.34 MB MOV)

Video S3 Transverse sections through time-series statistical parametric maps. Gray background is pre-injection MDA, while the colored overlays denote areas with increased intensity (P<0.00025) at 1 hr (green), 4 hr (red), 8 hr (yellow), and 24 hr (blue) compared to the preceding time point. Video shows consecutive sections in the transverse directions for the DAT knockout and wild-type cohorts. Slice locations are shown at the bottom right in millimeters with respect the brain surface. Scale bar  = 1 mm.
(5.69 MB MOV)

Video S4 Three dimensional rotations of statistical parametric maps. Three dimensional rotations of statistical parametric maps with Gray background is pre-injection MDA, while the colored overlays denote areas with increased intensity (P<0.00025) at 1 hr (green), 4 hr (red), 8 hr (yellow), and 24 hr (blue) compared to the preceding time point.
(9.44 MB MOV)
