# Supplementary figures and images for: Altered Neurocircuitry in the Dopamine Transporter Knockout Mouse Brain
Source: PLoS One. 2010 Jul 9;5(7):e11506. doi: 10.1371/journal.pone.0011506 (PMC2901340; doi:10.1371/journal.pone.0011506)

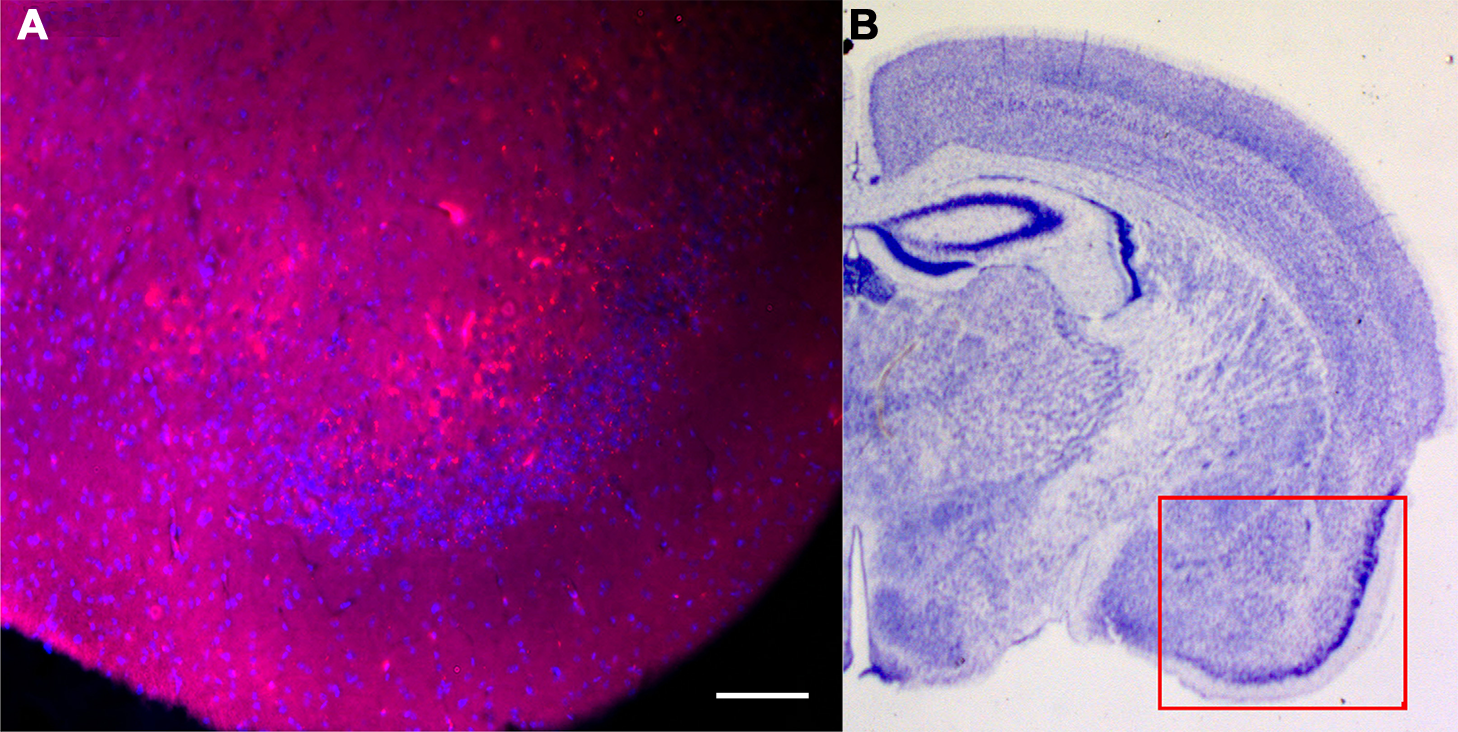

Supplement: Figure S1 — Rhodamine-dextran is transported to the amygdala in a wild type animal 16 days after injection in the PFC. The sections was mounted, stained with DAPI for nuclei and imaged for rhodamine and DAPI fluorescence. Alternate sections were Nissl stained. A) Rhodamine-dextran appears in the red channel in neurons of the amygdala and not in other areas in this part of the brain. The DAPI-stained nuclei are shown in blue. B) Nissl-stained section adjacent to the section shown in A) which overlaps the boxed area. These results demonstrate that the location of the injection site was appropriate for the introduction of tracer into the expected forebrain-midbrain pathway. The location of the rhodamine fluorescence is consistent with rhodamine-dextran having arrived via retrograde transport along the axons to the neuronal cell body. Scale bar = 100 µm. (3.23 MB TIF) [file pone.0011506.s001.tif]
